# New challenges for lung transplantation in the era of COVID-19

**DOI:** 10.1590/1516-3180.2022.140126082021

**Published:** 2022-01-17

**Authors:** Lucas Matos Fernandes, Paulo Manuel Pêgo-Fernandes

**Affiliations:** I MD. Attending Physician, Thoracic Surgery Service, Lung Transplant Group, Instituto do Coracao, Hospital das Clinicas HCFMUSP, Faculdade de Medicina, Universidade de Sao Paulo, Sao Paulo, SP, BR.; II MD, PhD. Full Professor, Thoracic Surgery Program, Instituto do Coracao, Hospital das Clinicas HCFMUSP, Faculdade de Medicina, Universidade de Sao Paulo, Sao Paulo, SP, BR; Director, Scientific Department, Associação Paulista de Medicina, São Paulo (SP), Brazil.

The COVID-19 pandemic caused by the severe acute respiratory syndrome coronavirus 2 (SARS-CoV-2) began in late 2019 and has caused immense numbers of hospitalizations and deaths worldwide. There have now been more than 190 million diagnosed cases and more than 4 million deaths, according to the Johns Hopkins coronavirus center.^1^Organ transplantation, like most other surgical procedures, and especially lung transplantation, has been extensively affected by the pandemic,^2^due to high rates of hospital occupation and chances of infection, and to reallocation of healthcare professionals. Patients on waiting lists have faced not only the natural morbidity and mortality of their underlying diseases, but also an additional risk of complications due to COVID-19. Furthermore, the diminished supply of donors and difficulty in ensuring that these donors are negative for coronavirus infection have decreased the number of transplantations performed, thus increasing the mortality rate among waitlisted patients.

On the other hand, as already seen in other pandemics such as influenza, patients with severe acute respiratory syndromes due to current viral respiratory infections usually evolve without the need for lung transplantation, either because the catastrophic outcome of death occurs or because the pulmonary condition improves. However, during the COVID-19 pandemic, cases of chronification of acute lung transplantation cases have been reported worldwide. Most of these cases consisted of patients with lung disease for more than four to six weeks from which they had not recovered, but who did not have any other organic dysfunctions.

In mid-2020, after the first reports of transplantations in this type of patient were published, the Toronto group presented an article containing 10 guidelines to be considered in patients before indicating lung transplantation:^3^1. Age (< 65 years); 2. Pulmonary dysfunction alone; 3. Long enough time for pulmonary recovery (something around four to six weeks at least); 4. Radiological evidence of irreversibility of the pulmonary condition; 5. The patient is awake and aware enough for discussion about transplantation; 6. Suitability for rehabilitation physiotherapy; 7. Meeting the usual criteria for lung transplantation (e.g. with regard to body mass index, drug addiction, smoking, etc.); 8. Polymerase chain reaction (PCR)-negative for SARS-CoV-2 in the lower respiratory tract; 9. The patient is at an experienced transplantation center; and 10. The center has extensive access to donors and there is low mortality on the waiting list.

In view of the low number of transplantations performed at the beginning of the pandemic, and the need for transplantations in this emerging population, the São Paulo State Technical Chamber for Thoracic Organs met to define criteria for patients to be evaluated and included in a list of those with severe acute insufficiency secondary to COVID-19 and, in April 2021, the criteria presented in Annex 1were agreed.

Based on this guidance, a series of evaluations was started, among patients institutionalized in the hospital complex of Hospital das Clínicas, Faculdade de Medicina, Universidade de Sao Paulo (HC-FMUSP), and among external patients (via telemedicine, who were then, if they fulfilled the minimum criteria, transferred to InCor/HC-FMUSP). Through this process, five institutionalized patients were analyzed: two were placed on the waiting list and then received transplants. Six external patients were evaluated: two were transferred, of whom one was waitlisted and received a transplant and the other, after evaluation and rehabilitation, presented clinical improvement and is on course for hospital discharge without the need for transplantation. Much of this effort can be attributed to the intensive participation of the multidisciplinary team, with special mention for the physiotherapy, nutrition and intensive care unit (ICU) staff.^4^

Returning to surgical practice and the processes of donor selection, organ harvesting and operative tactics, to undertake transplantation in the midst of the pandemic, brought to light several points of note. In selecting recipients, in addition to the usual guidelines or ideal criteria, extended criteria were applied (Table 1).^5^Thus, a routine was created that included investigation of the clinical history of previous disease and exposure to contacts, a negative PCR test for SARS-CoV-2 and chest tomography without presentation of the infiltrates of ground-glass appearance that are characteristic of cases of COVID-19. In this manner, it was sought to ensure safety for the transplantation team, the recipient and the entire hospital to which the organ was sent, and thus to avoid occurrences of contamination of the team through inadvertent use of lungs infected with COVID-19, as in some published reports.^6^However, the procedure for extracting the lung block was done in the usual way, consisting of evaluation, perfusion and removal.

**Table 1:** Ideal and extended criteria for donor selection

Ideal criteria	Extended criteria
Age < 55 years	Age > 55 years
Normal radiograph	Abnormal radiograph
**PaO**_2_**> 300 (PEEP 5; FiO**_2_100%)	PaO_2_< 300 (PEEP 5; FiO_2_100%)
Without thoracic trauma	With thoracic trauma
Sputum Gram stain: absence of organisms	Presence of microorganisms in tracheal secretions
No smoking	Smoking > 20 pack-years
Absence of purulent secretions at bronchoscopy	
No aspiration or sepsis	

Adapted from Orens JB et al.^5^

PaO_2_= arterial partial pressure of oxygen; PEEP = positive end-expiratory pressure; FiO_2_= fraction of inspired oxygen.

On the recipient side, there were also some points of note regarding the implantation surgery. Most patients who were awaiting lung transplantation due to severe acute respiratory syndrome consequent to COVID-19 were in situations of receiving circulatory extracorporeal membrane oxygenation (ECMO).^7–9^This was implemented initially for ventilatory disorders, but ultimately due to pulmonary hypertension associated with low compliance. These patients were monitored by means of invasive radial and femoral blood pressure measurements, bispectral index (BIS), Swan-Ganz, pulse oximeter, cardioscopy and transesophageal echocardiography.

In our experience, we chose to implement central venoarterial ECMO as intraoperative care, while maintaining peripheral venovenous ECMO, because the membranes had already been in use for more than three weeks. In this manner, contamination within the operative field was avoided, as described in an article published in this edition of the São Paulo Medical Journal.^10^Another complicating factor was that the patients very commonly presented pneumothorax and firm pleuro-pulmonary adhesions, and even drains were present. We chose to start the surgery on the side most affected by the disease, as is usually done, using the side with lower perfusion. Friable and hypervascularized lymph nodes of greater numbers and size were found in both hila. Reperfusion of the lungs was done slowly and gradually and the pressure of the pulmonary arteries was controlled by decreasing or increasing the circulatory assistance during the release of clamps from the pulmonary artery and left atrium. Throughout the transplantation, a cell saver was used. However, there was a need for blood transfusion and for replacement of fibrinogen and prothrombic factors. At the end of the transplantation, in the event of preserved biventricular function, we chose to remove the central venoarterial assistance and maintain the ECMO circuit circulating with saline solution, after total or partial return of the blood from the circuit. If it was not possible to fully return the blood, the rest was aspirated to the cell saver for reuse of the red blood cells. If, at the end of transplantation, there was a need for circulatory assistance due to ventricular dysfunction, we chose to switch to peripheral cannulation of the femoral artery using a thin cannula (15Fr), for partial assistance until recovery of cardiac function.

Before closing the chest wall, an extensive review of hemostasis was performed and pleural drains were placed in both pleurae, in line with the usual practice, one anteriorly and one posteriorly. During this period, tests on ventilatory autonomy and on shutdown of venovenous ECMO were performed, such that there was no entry of gas into the membrane via ECMO during this period. If the lungs maintained good oxygenation and exchange of carbon dioxide, the peripheral ECMO was also removed at the end of the surgery and the patient was sent to the intensive care unit for postoperative care.

Despite the replacement of the target organ of COVID-19, this type of patient remained critical. Early dialysis was necessary in operated cases, with maintenance of broad-spectrum antibiotics and antibiotics directed to germs previously identified and cultured from donor materials such as bronchoalveolar lavages. Tracheostomy was an important facilitator, with the aim of waking the patient and maintaining the rehabilitation that had been started preoperatively.

Lung transplantation in the midst of the pandemic proved to be a major challenge. This related both to searching for viable donors and to avoiding mortality among severely ill patients on the waiting list or among acute patients who progressed to a chronic form without other important organic dysfunctions, to allow them access to this type of treatment.

With the increase in mass vaccination and a better understanding of COVID-19, its treatments and cure, we believe that transplantation for acute patients due to this disease is a fleeting issue. Nevertheless, the lessons learned in the midst of this chaos will not be lost. The use of ECMO in patients with severe acute respiratory syndrome, which had already become part of the arsenal of large centers, will increasingly become used routinely. Although there is still a lack of exact criteria regarding which patients would have the best outcome from COVID-19 through use of this tactic, it has certainly marked its presence in relation to care for critical respiratory patient and for intraoperative support, as had already been seen in relation to patients with pulmonary hypertension.

We believe that transplantation plays a role in treatment for very well-selected acute lung patients who have evolved to chronic disease without presenting dysfunction of other organs. There is still a lack of studies and analyses on datasets larger than the case series seen over recent months, for enabling better definition of indications and outcomes. Until then, careful selection of patients and implementation of transplantation in large specialized centers and use of ECMO and rehabilitation are necessary in order to try to optimize the results and not waste lungs.

## Annex 1 Criteria established by the São Paulo State Technical Chamber for Thoracic Organs for inclusion in transplantation waiting lists of patients with severe acute insufficiency secondary to COVID-19, in April 2021

**Table:**

1. Polymerase chain reaction (PCR) negative for COVID-19 in samples, among which at least one should be from the lower respiratory tract;
2. Age less than 65 years (and ideally between 18 and 50 years);
3. Irreversibility of the clinical condition for more than six months;
4. Body mass index prior to hospitalization of between 17 and 27;
5. Hemodynamic stability;
6. Absence of active bacterial or fungal infections;
7. Patient awake and understanding that he or she will be evaluated for transplantation, and in agreement with undergoing this transplantation. Presence of an adequate caregiver who has been assessed by the nursing and psychology teams;
8. Approval from social services and absence of drug addiction, including smoking;
9. Suitable for active rehabilitation (minimum strength level of grade 3);
10. Ejection fraction greater than 50%. Transesophageal echocardiogram without showing any vegetations or anatomical or functional abnormalities;
11. Absence of coronary obstructions that cannot be treated percutaneously;
12. Absence of other organ dysfunctions;
13. Signing of a consent statement;
14. Autonomy is to be retained by the transplantation team for contraindicating the transplantation according to other clinical information that is collected and evaluated by the multiprofessional team.
